# Regulation of DNA methylation on *EEF1D* and *RPL8* expression in cattle

**DOI:** 10.1007/s10709-017-9974-x

**Published:** 2017-06-30

**Authors:** Xuan Liu, Jie Yang, Qin Zhang, Li Jiang

**Affiliations:** 1 National Engineering Laboratory for Animal Breeding, Beijing, China; 2 Key Laboratory of Animal Genetics, Breeding and Reproduction, Ministry of Agriculture of China, Beijing, China; 3 College of Animal Science and Technology, China Agricultural University, Beijing, 100193 China

**Keywords:** Methylation, *EEF1D*, *RPL8*, Protein expression, Dairy cattle

## Abstract

Dynamic changes to the epigenome play a critical role in a variety of biology processes and complex traits. Many important candidate genes have been identified through our previous genome wide association study (GWAS) on milk production traits in dairy cattle. However, the underlying mechanism of candidate genes have not yet been clearly understood. In this study, we analyzed the methylation variation of the candidate genes, *EEF1D* and *RPL8*, which were identified to be strongly associated with milk production traits in dairy cattle in our previous studies, and its effect on protein and mRNA expression. We compared DNA methylation profiles and gene expression levels of *EEF1D* and *RPL8* in five different tissues (heart, liver, mammary gland, ovary and muscle) of three cows. Both genes showed the highest expression level in mammary gland. For *RPL8*, there was no difference in the DNA methylation pattern in the five tissues, suggesting no effect of DNA methylation on gene expression. For *EEF1D*, the DNA methylation levels of its first CpG island differed in the five tissues and were negatively correlated with the gene expression levels. To further investigate the function of DNA methylation on the expression of *EEF1D*, we collected blood samples of three cows at early stage of lactation and in dry period and analyzed its expression and the methylation status of the first CpG island in blood. As a result, the mRNA expression of *EEF1D* in the dry period was higher than that at the early stage of lactation, while the DNA methylation level in the dry period was lower than that at the early stage of lactation. Our result suggests that the DNA methylation of *EEF1D* plays an important role in the spatial and temporal regulation of its expression and possibly have an effect on the milk production traits.

## Background

Milk production traits are the most important traits in dairy cattle. In the past decades, many studies have been carried out to reveal the genetic basis of milk production traits (Zhang et al. 1998; Farnir et al. 2002; Ashwell et al. 2004; Jiang et al. 2010; Mai et al. 2010) and a lot of candidate genes or QTL affecting milk yield and milk components have been reported (Viitala et al. 2006; Winter et al. 2002; Wang et al. 2013). Along with the development of cost-effective “omics” technology, many powerful tools are being used to identify functional genes (mutations) and their regulatory mechanisms recently, such as genome-wide association studies (GWAS) (Jiang et al. 2010; Mai et al. 2010; Meredith et al. 2012; Cole et al. 2011) and gene expression profiles (Wickramasinghe et al. 2012; Connor et al. 2013; Singh et al. 2013; Cui et al. 2014). Although these findings provide new insights into genetic basis of milk production traits, the underlying mechanism of potential candidate genes have not yet been clearly understood.

In our previous GWAS study in Chinese Holstein cattle, 105 significant SNPs associated with milk yield and composition traits were identified (Jiang et al. 2010). In the followed study, we used the target enrichment technology and next generation sequencing (NGS) to assess the candidate regions implicated by significant SNPs in our GWAS and then we conducted association analysis for 200 important variants revealed by NGS in a new dairy cattle population (Jiang et al. 2014). As a result, a total of 66 significant SNPs involved in 53 genes were identified. Of these, one SNP located in the promoter region of *EEF1D* showed strong association with milk yield, fat percentage and protein percentage with *P* values of 9.23E-06, 2.07E-15 and 1.26E-07, respectively (Jiang et al. 2014). Furthermore, one SNP in the promoter region of *RPL8* was also identified to be associated significantly with milk yield, protein yield and fat percentage with *P* values of 2.62E-07, 6.63E-05 and 2.26E-15, respectively (Jiang et al. 2014). In addition, we investigated the mRNA expression of 20 significant candidate genes in different tissues in dairy cattle and most of them showed higher expression in mammary gland than in other tissues. In particular, both *EEF1D* and *RPL8* had the highest expression in mammary gland among the 20 genes. Therefore, *EEF1D* and *RPL8* were considered as two promising candidate genes for milk production traits in dairy cattle.

*EEF1D* encodes a subunit of the elongation factor-1 complex, which is responsible for the enzymatic delivery of aminoacyl tRNAs to the ribosome and functions as a guanine nucleotide exchange factor (Ogawa et al. 2004). *RPL8* encodes the 60S ribosomal protein L8, which is a component of the ribosomes 60S subunit. This protein belongs to the L2P family of ribosomal proteins and is located in the cytoplasm. It has been reported that the mRNAs of components of the 60S subunit were highly abundant in bovine mammary and contributed to protein synthesis (Bionaz and Loor 2011). Both of these two genes are located on chromosome 14, which has been reported to harbor major QTLs for milk production traits, especially for fat percentage (Winter et al. 2002; Cole et al. 2011; Coppieters et al. 1998; Kaupe et al. 2007).

Methylation of CpG islands in the promoter region of genes has been widely known to be involved in a variety of biology processes and complex diseases, such as embryo development (Smith et al. 2012; Smallwood et al. 2011) and tumorigenesis (Ronneberg et al. 2011; Dedeurwaerder et al. 2011). Recently, studies on DNA methylation profiling across the genome are increasing. Different genome-wide DNA methylation maps have been reported in many distinct tissues and organisms, such as human (Slieker et al. 2013; Davies et al. 2012; Day et al. 2013), chimpanzee (Pai et al. 2011) and rat (Hon et al. 2013). Many epigenetic studies revealed that the aberrant DNA methylation of CpG islands in the promoter regions results in inactivation of genes and plays an important role in tumor progression (Park et al. 2011; Cai et al. 2011). Differential CpG island methylation contributes to the gene expression by influencing transcription factors binding, altering genomic structure and regulating the microRNA expression levels (Jones and Liang 2009; Herman and Baylin 2003; Chellappan et al. 2010). However, few epigenetic modification studies have been reported for milk production traits in dairy cattle.

In this study, we analyzed the DNA methylation pattern of the CpG islands in the promoter regions of *EEF1D* and *RPL8*. Our results revealed that the DNA methylation level of one CpG island of *EEF1D* was significantly negatively correlated with the expression level of EEF1D in different tissues and different periods in dairy cattle. Our study provides more information on epigenetics in dairy cattle.

## Materials and methods

### Animals and tissue sample collection

Three lactating Chinese Holstein cows were selected from the Beijing Sanyuan Dairy Farm Center. All of them were fed in a standard environmental condition. Five tissue samples (heart, liver, mammary gland, ovary and muscle) from each individual were collected within 30 min after slaughter and stored at liquid nitrogen. The whole procedure for collection of tissue samples of all animals was carried out in strict accordance with the protocol approved by the Animal Welfare Committee of China Agricultural University (Permit number: DK996).

### Western blotting analysis

Western blotting analysis was performed to detect the protein expression levels of *EEF1D* and *RPL8* in different tissues. Total proteins were extracted from the five tissues samples (heart, liver, mammary gland, ovary and muscle) of three different individuals. A total of 60 µg of proteins were separated on 10% SDS–PAGE, and then transferred to PVDF membranes (BIO-RAD). After blocked with 5% skim milk for 1.5 h at room temperature, the membranes were incubated with the primary antibody at 4 °C over night (*EEF1D*: Abcam; *RPL8*: Santa Cruz Biotechnology; *GAPDH*: Santa Cruz Biotechnology), and then further incubated with corresponding HRP-conjugated secondary antibodies (Sigma) for 1 h at room temperature. The labeled bands were visualized by using the ECL kit (BIO-RAD). The Photoshop software was used to quantify the relative expression levels.

### Methylation analysis

Genomic DNA was extracted from the five tissue samples of the three cows using the commercial kit (Tiangen Biotech, Beijing, China). The quantity and quality of DNA were measured using NanoDrop^TM^ND-2000c Spectrophotometer (Thermo Scientific, Inc.). The DNA was chemically modified by sodium bisulfate to convert all unmethylated cytosines to uracils while leaving methylcytosines unaltered using the EZ DNA Methylation-Gold Kit (Zymo Research, CA, USA). CpG islands in the promoter region were detected by using the CpG Plot web-tool (http://www.ebi.ac.uk/Tools/emboss/cpgplot/). For these regions, three pairs of primers were designed to carry out Methylation-Specific PCR (Table 1). After amplification, the PCR products were cloned into a pBLUE-T vector. The plasmid was then used to transform TOP competent cells. Ten colonies per sample were chosen randomly for sequencing.

**Table 1:** Primers for methylation-specific PCR for the CpG islands in *EEF1D* and *RPL8*

CpG island	Forward	Reverse	CpG sites^a^	Length^b^
*EEF1D*-1	5′-GGAGGATAAGTAGAAGTATGGGGAA-3′	5′-TAATAACAAACCACCTAACTCC-3′	32	326
*EEF1D-*2	5′-GGGAGGTGTGGTTGGAGAAAT-3′	5′-TCTCAAACTAAACATAAATAAACCC-3′	37	318
*RPL8*-1	5′-GTTTTTTTTAGAGTAGTTAGGGTTTTTAG-3′	5′-TTCCACCTCCTCTTTTACTAACTCC-3′	24	211

^a^The number of CpG sites included in the island

^b^The length of the CpG island

## Results

### Analysis the promoter region of *EEF1D*

**Fig. 1:**
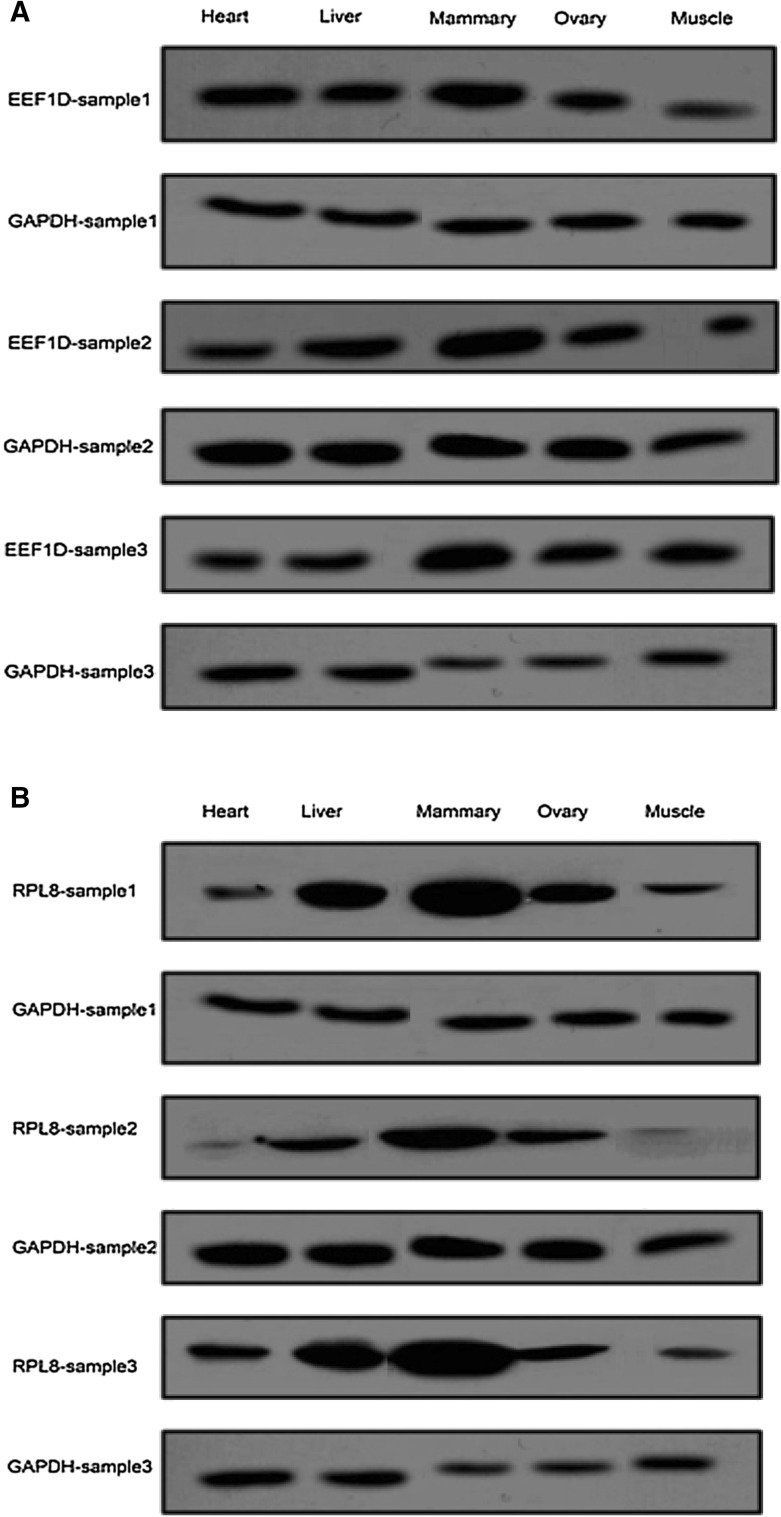
Relative protein expression of *EEF1D* (**a**) and *RPL8* (**b**) in five tissues of three lactating cows detected by Western blotting. *GAPDH* was used as a control

We first analyzed the expression of the EEF1D protein in the five tissues (heart, liver, mammary gland, ovary and muscle) of the three cows by using Western blotting. The results showed about two- to three-fold higher expression level in mammary gland than in other tissues (Fig. 1a), which is well in accordance with the mRNA expression of *EEF1D* in these tissues in our previous study (Jiang et al. 2014).

**Fig. 2:**
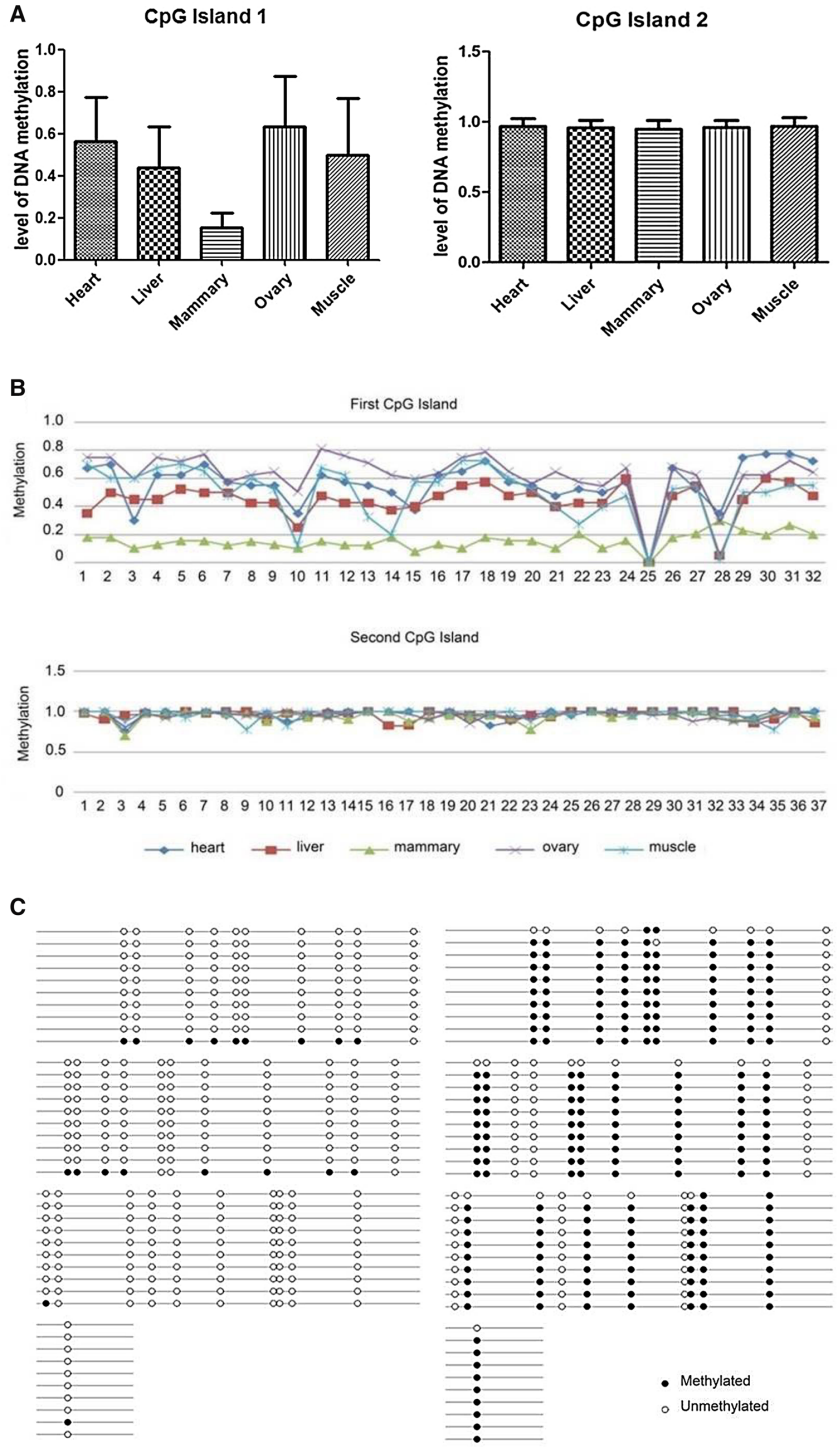
Methylation analysis of the two CpG islands in the promoter region of EEF1D in five tissues of three lactating cows. **a** Methylation patterns of the two CpG islands. *Error bars* represent the standard deviation. **b** Methylation profiles of all sites of the two CpG islands. **c** Methylation status of the 32 sites of the first CpG island in mammary gland (*left*) and muscle (*right*) of one cow. Ten independent PCR product clones were demonstrated for each *lines. Solid dot*, methylated CpG dinucleotide; *circle*, unmethylated GpG dinucleotide

Since no significant SNPs were found in the coding region of *EEF1D* in our previous association studies, we paid our attention to the promoter region. Two CpG islands (−2770 to −2444 bp and −2126 to −1808 bp upstream from ATG, respectively) were detected in the promoter region. The methylation status of the two CpG islands were quantitatively measured in five different tissues (heart, liver, mammary gland, ovary and muscle) of the three cows used for Western blotting analysis. For the first CpG island, the methylation status varied remarkably in different tissues (Fig. 2a). All the three individuals displayed significantly lower methylation level in mammary gland than in other tissues. The proportion of the methylated alleles in mammary gland was very low with an average of 16%, while it was 44–64% in other tissues (Fig. 2c). However, the methylation levels of the second CpG island were almost the same in all tissues and the proportion of methylated alleles reached nearly 100% (Fig. 2a). The methylation rates for each CpG site of the two CpG islands were also calculated for the three individuals and are displayed in Fig. 2b. The methylation levels in the first CpG island were well in accordance with the expression levels of *EEF1D* in different tissues, i.e., the lower the methylation level, the higher the protein expression levels, suggesting that the expression of *EEF1D* is regulated by the first CpG island methylation in its promoter.

### Analysis the promoter region of *RPL8*

We also analyzed the protein expression of *RPL8* in the five different tissues of the three cows by using Western blotting. We observed three- to seven-fold higher expression of the *RPL8* protein in mammary gland than in other tissues (Fig. 1b), which is well in accordance with the mRNA expression of *RPL8* in these tissues in our previous study (Jiang et al. 2014).

Just like the situation in *EEF1D*, no significant SNPs were detected in the coding regions of *RPL8* in our previous association studies, we focused our attention on its promoter region again. Only one CpG island (−252 to −41 upstream from ATG) was detected in the promoter region. However, the methylation analysis showed that there was no difference in methylation level among the five tissues. Besides, all the CpG sites of this island were unmethylated in the five different tissues. These results suggest that the differential expression of *RPL8* in different tissues is not regulated by DNA methylation of the CpG island in *RPL8* promoter in dairy cattle.

## Discussion

Following our previous GWAS based on bovine 50k SNP array (Jiang et al. 2010), target region association analysis based on targeted re-sequencing, and mRNA expression analysis of candidate genes, we performed protein expression and methylation analysis for two strong candidate genes, *EEF1D* and *RPL8*, in this study. Western blotting analysis showed that the expression of the EEF1D and RPL8 proteins were well in accordance with their mRNA expression, suggesting that EEF1D and RPL8 may play an important role in milk production traits in dairy cattle. Moreover, both of these two genes are located on chromosome 14 and *EEF1D* gene located a mere 806 kb away from *RPL8*. According to the gene annotation, RPL8 is a component of the ribosome 60S subunit. EEF1D encodes a subunit of the elongation factor-1 complex, which is responsible for the enzymatic delivery of aminoacyl tRNAs to the ribosome and functions as a guanine nucleotide exchange factor. EEF1D very likely participates in the binding of aminoacyl-tRNA at the ribosomal 60S subunit interface. Thus, there might be an interaction effect between these two genes. In addition, it has been reported that both *EEF1D* and the ribosome 60S were highly expressed in the mammary tissue of lactating cows (Jiang et al. 2014; Bionaz and Loor 2011). Therefore, both *EEF1D* and *RPL8* play important roles in milk production, either separately or interactively. Considering no significant variants in the coding regions were detected, we focused our attention on their promoter regions, where SNPs with significant effects on milk fat percentage were identified.

DNA methylation status in promoter region of a gene has been proved to play important roles in regulation of gene expression (Zhang et al. 2012; Heyn and Esteller 2012; Heyn et al. 2013). In particular, hypermethylation of the promoter region of a gene could effectively silence its transcription (Jandrig et al. 2004; Yang et al. 2009; Misawa et al. 2013). Therefore, we investigated the methylation patterns of *EEF1D* and *RPL8*. For *EEF1D*, we found two CpG islands in its promoter region, and the methylation level of the first CpG island in mammary gland was much lower than in other tissues, which was well in accordance with the mRNA and protein expression of *EEF1D* in these tissues, i.e., the higher (lower) expression level was corresponding to the lower (higher) methylation level, while the methylation levels of the second CpG island were almost the same in all tissues. These results suggest that the expression of *EEF1D* is regulated by the methylation status of the first CpG island in its promoter region. For *RPL8*, we found only one CpG island in its promoter region. However, no difference in methylation level among different tissues was detected, suggesting that the differential expression of *RPL8* in different tissues is not relevant to the methylation status in its promoter region.

**Fig. 3:**
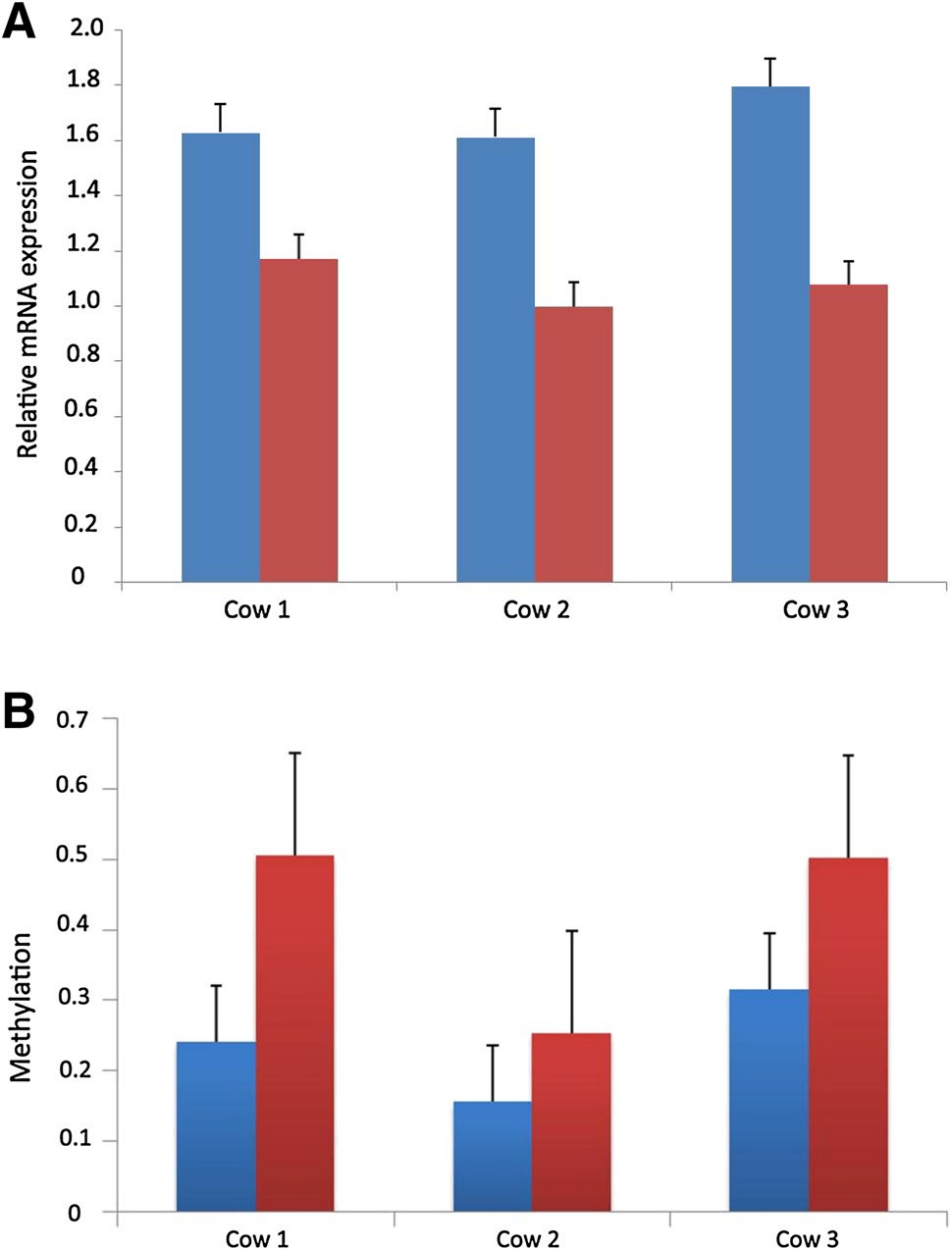
Relative mRNA expression (**a**) and methylation level (**b**) in blood of three cows at dry (*blue*) and lactating (*red*) periods. *Error bars* represent the standard deviation

To further confirm the regulation effect of methylation at the first CpG island in the promoter region of *EEF1D* on its expression, we collected blood samples of another three cows at early stage of lactation (15 days in milk) as well as in dry period (30 days before calving) and analyzed the mRNA expression of *EEF1D* and the methylation status of this CpG island in blood in the two periods. As a result, the mRNA expression in the dry period was higher than that at the early stage of lactation in all the three cows (Fig. 3a), while the methylation level in the dry period was lower than that at the early stage of lactation (Fig. 3b). These results support the methylation is negative association with the expression of *EEF1D*.

The function of a gene is closely related to its specifically spatial and temporal expression. Many previous studies reported that a large amount of genes were down-regulated in lactation stage compared with in non-lactation period in bovine mammary gland tissue (Finucane et al. 2008; Gao et al. 2013). In our previous study (Yang et al. 2016), we detected differential expression of genes by using RNA-seq in milk samples of two groups of cows with extremely high and low 305-day milk yield, milk fat yield and milk protein yield, respectively, at day 10 (early stage of lactation) and day 70 (peak stage of lactation) after calving. Our results indicated that most of the differentially expressed genes showed lower expression in the cows of group of high yield as well as at the peak stage of lactation. This implied the high milk production is associated with down-regulation of majority of genes. The expression of *EEF1D* in blood of cows in dry period and at the early stage of lactation showed the similar pattern, i.e., higher expression in dry period than at the early stage of lactation. It is reasonable to expect that this pattern would also be observed in mammary gland. This suggests that the expression of *EEF1D* is also related to the on-set of lactation and is regulated by the methylation at its first CpG island.

Some previous studies reported that epigenetic regulation might be involved in oocyte development, spermatogenesis and fat deposition (Diederich et al. 2012; Luo et al. 2013; Liu et al. 2011; Magee et al. 2010). Our data implied that epigenetic modification changed the expression of some important genes, which were significantly associated with milk production trait. Currently, genomic selection (GS) is the widely used in dairy cattle breeding. The theory of GS assumes that differences in DNA sequence lead to genetic differences between animals. However, there is evidence for epigenetic differences between individuals. These epigenetic differences are associated with changes in the expression of genes. In cattle, it has been reported that the additive effects of SNPs only explain 32–80% of genetic variance (Goddard ME and Whitelaw 2014). Although it is difficult to answer the relationship between methylation status and SNPs, it would be worthwhile to know more epigenetic information of major genes in cattle genome and to include these information in genetic evaluation approaches in the future to increase selection efficiency in cattle populations.

## Conclusion

In mammals, tissue-specific DNA methylation patterns were established only in pigs (Li et al. 2012a, b). In this study, we found that the DNA methylation of *EEF1D* likely plays an important role in its transcriptional regulation and may have a severe effect on milk production traits in dairy cattle. Our results contribute to the knowledge of epigenetic effects in dairy cattle and promotes a better understanding of the global genetic architecture of milk production traits.

## Abbreviations

EEF1D: Eukaryotic translation elongation factor 1 delta
RPL8: 60S ribosomal protein L8
GWAS: Genome-wide association study
NGS: Next generation sequencing
SNP: Single nucleotide polymorphism
